# Efficacy and safety of Bu Jing Yi Shi tables for high myopia: A protocol of systematic review and meta-analysis

**DOI:** 10.1097/MD.0000000000024130

**Published:** 2021-01-22

**Authors:** Jie Ma, Linzhi Li, Ya Mo

**Affiliations:** a Chengdu University of Traditional Chinese Medicine; b Hospital of Chengdu University of Traditional Chinese Medicine, Chengdu, China.

**Keywords:** Bu Jing Yi Shi tables, high myopia, meta-analysis, protocol, systematic review

## Abstract

**Background:**

High myopia is a kind of ametropia with diopter more than −6.00D or axial length ≥26 mm. With the change of the modern environment, the incidence rate is increasing year by year. At present, the pathogenesis of high myopia is not clear. Some current studies indicate that it may be related to the environment and genetics. A Chinese patent medicine named Bu Jing Yi Shi Tablets (BJYST) has many functions including anti-oxidation, expansion of blood vessels, anti-inflammatory, immune regulation, inhibition of retinal photoreceptor cell apoptosis, and promotion of retinal repair. A large number of existing studies have shown that this prescription can relieve the clinical manifestations of high myopia and its complications, but its true efficacy and safety are still unclear. To certify this point, a protocol for a systematic review and meta-analysis of BJYST for high myopia will be performed.

**Methods and analysis:**

Articles that have been identified by electronically searching of 9 English and 5 Chinese databases from their inception to December 4, 2020 will be incorporated into the study. This study only adopts Chinese and English. Two researchers will take charge of completing the selection of research, the extraction of data as well as the assessment of research quality independently. The primary outcomes will be an average change in refractive error measured in diopters and an average change in axial length measured in millimeters. Data analysis will be performed via the RevMan 5.3 software, and Grading of Recommendations Assessment, Development, and Evaluation (GRADE) will help to assess the evidence level.

**Results:**

The results of this study will be published in a peer-reviewed journal.

**Conclusion:**

This study will conclude whether BJYST is safe and effective in treating high myopia on the basis of evidence-based medicine.

**Registration:**

The Open Science Framework (OSF) registration number is osf.io/dpk5b.

## 1 Introduction

High myopia is a kind of ametropia with diopter more than −6.00D or axial length ≥26 mm. With the change of the modern environment, the incidence rate is increasing year by year.^[1]^ Studies have shown that by 2050, the number of patients with high myopia in the world may reach 1 billion, which will bring huge economic expenditures.^[2–4]^ High myopia is often accompanied by a series of fundus changes, including posterior scleral staphyloma, myopic maculopathy (such as fundus atrophy, choroidal neovascularization, myopic macular splitting, etc.) and peripheral retinal degeneration. In addition, complications of high myopia may cause irreversible vision impairment, such as glaucoma, cataracts, retinal detachment, and macular degeneration, etc.^[3–5]^ At present, the pathogenesis of high myopia is not clear. Some current studies indicate that it may be related to the environment and genetics.^[6]^ It is worth noting that the occurrence of high myopia seems to be obviously related to race, age, and gender.^[7–9]^

The purpose of treatment for high myopia is to correct refractive errors and control eye axis elongation. The main methods are conservative and surgical treatment. Conservative treatment including the use of atropine, enhancing outdoor activities, reducing near work, and so on, can play a therapeutic role to a certain extent.^[10,11]^ So far, there are many surgical methods, such as posterior scleral reinforcement, combining femtosecond thin- flap and LASIK with the Triple-A profile, and Single-step Transepithelial photorefractive keratectomy, etc.^[12–14]^ Although studies have shown that surgical intervention can inhibit vision loss and axial elongation, related side-effects may also occur. For example, photophobia, overcorrection, allergic conjunctivitis, and Higher-Order Aberration increased, etc.^[13–15]^ The symptoms of these adverse reactions would be relieved by treatment in the later stage, but some patients questioned the effect of the treatment and gave up further treatment, eventually, the development of the disease will cause irreversible damage.^[3–5]^

Chinese herbal medicine is one of the most important parts of Traditional Chinese Medicine(TCM), which has been used for thousands of years. Bu Jing Yi Shi Tablets (BJYST) is Chinese patent medicine which derived from the classic prescription Zhujing Wan in “The Six Classics of Ophthalmology” by Chen Dafu, a well-known expert in Chinese ophthalmology. According to recent pharmacological studies, It's herbal ingredients has antioxidant, vasodilator, anti-inflammatory, immune regulation, inhibit retinal photoreceptor cell apoptosis, and promote retinal repair.^[16–21]^ A large number of existing studies have shown that this prescription can relieve the clinical manifestations of high myopia and its complications, but its true efficacy and safety are still unclear.^[22–27]^ To certify this point, a protocol for a systematic review and meta-analysis of BJYST for high myopia will be performed.

## 2 Objectives

To establish a systematic and comprehensive approach for locating the evidence, a review and meta-analysis will be used to whether the BJYST is effective and safe in the treatment of high myopia.

## 3 Methods

### 3.1 Study registration

The protocol has been registered on OSF platform and the registration number is osf.io/dpk5b. The protocol was written following the statement guidelines of Preferred Reporting Items for Systematic Reviews and Meta-Analyses Protocols (PRISMAP).^[28]^

### 3.2 Inclusion and exclusion criteria for study selection

Inclusion criteria: all randomized controlled trials (RCTs) about BJYST to treat high myopia. The review only adopts the language of English and Chinese.

Exclusion criteria: Non-RCTs, quasi-RCTs, case series, reviews, and animal studies.

### 3.3 Types of participants

Patients clinically diagnosed with high myopia will be studied. Participants in this study will not have gender, age, ethnicity, job, or education restrictions.

### 3.4 Types of interventions

#### 3.4.1 Experimental interventions

The experimental group will include patients receiving treatment of BJYST. The treatment frequency and duration are not restricted.

#### 3.4.2 Control interventions

The Control group will include patients receiving control interventions including placebos or drugs that have been proven effective in the treatment of high myopia.

### 3.5 Types of outcome measures

#### 3.5.1 Primary outcomes

The primary outcomes will be an average change in refractive error measured in diopters and an average change in axial length measured in millimeters.

#### 3.5.2 Secondary outcomes

Secondary outcomes include Change of the visual field, Change of retinal light sensitivity, Change of contrast sensitivity function, and Change of electroretinograms b-wave (ERGb) amplitude.

### 3.6 Search methods for study identification

**Figure 1:**
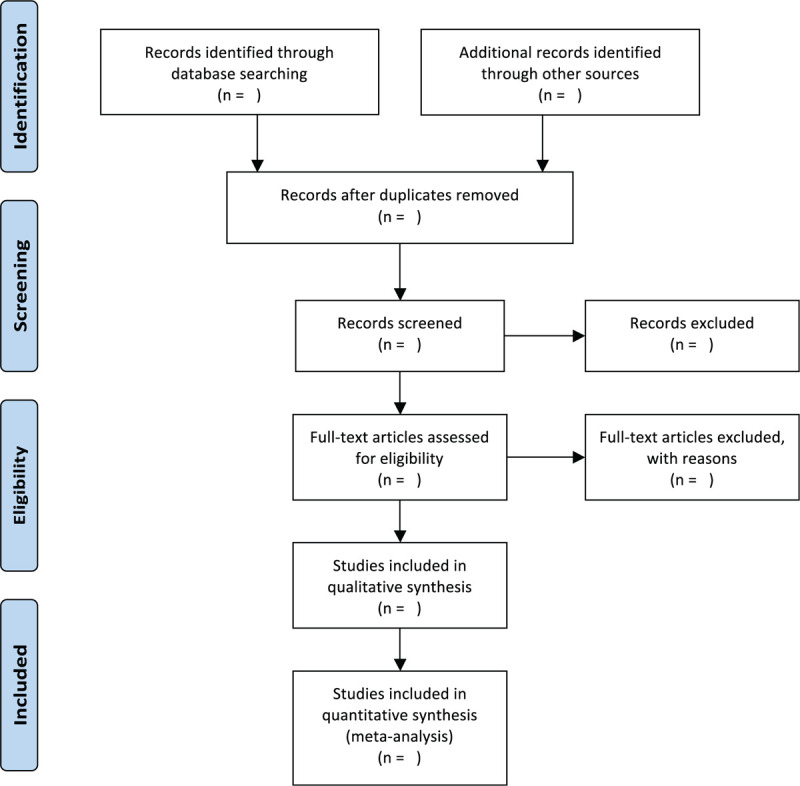
The research flowchart.

Electronic searching will focus on databases of the Cochrane Library, MEDLINE, EMbase, AMED, Nature, Science Online, PubMed, WorldSciNet, World Health Organization International Clinical Trials Registry Platform (ICTRP), the Wanfang Database and China Biology Medicine Disc, and China National Knowledge Infrastructure, the Chongqing VIP Chinese Science and Technology Periodical Database, with the temporal from the inception of database to December 4, 2020. For ongoing RCTs, researchers will contact the trial author to obtain the latest clinical data. Besides, studies associated with the review will be identified by evaluating related conference proceedings. The research flowchart is shown in Figure 1.

A search strategy of the combination of text words and Medical Subject Headings (MeSH) terms will be adopted. Appendix (Table 1) displays a detailed search strategy for the PubMed database. According to the difference of databases, keywords may be combined with free words and a comprehensive search will be performed.

**Table 1:** Search strategy for the PubMed database.

Number	Search Terms
1	High myopia. Mesh.
2	High myopia. ti, ab.
3	1 or 2
4	Bu Jing Yi Shi Tables. Mesh.
5	Bu Jing Yi Shi Tables. ti, ab.
6	Bu Jing Yi Shi Table. ti, ab.
7	Bu-Jing-Yi-Shi Tables. ti, ab.
8	Bu-Jing-Yi-Shi Table. ti, ab.
9	Bujingyishi Tables. ti, ab.
10	Bujingyishi Tables. ti, ab.
11	BJYST. ti, ab.
12	4 or 5–11
13	Randomized controlled trial. Mesh.
14	Randomized controlled trial. pt
15	Controlled clinical trail. Mesh.
16	Controlled clinical trail. pt
17	Clinical trial. pt
18	Random allocation. Mesh.
19	Random allocation. ti,ab
20	Randomly.ti,ab
21	Randomized. ti,ab
22	Double-blind method. Mesh.
23	Double-blind method. ti,ab
24	Single-blind method. Mesh.
25	Single-blind method. ti,ab
26	13 or 14–25
27	3 and 12 and 26

### 3.7 Data collection and analysis

#### 3.7.1 Study selection

After the independent information extraction from literature included in the study, two researchers will input the extracted information into a unified statistical table of data. Ineligible studies as well as duplicate records will be first eliminated, followed by a review of the full text of those eligible ones for confirming their compliance to the abovementioned inclusion criteria. If the two researchers cannot come to an agreement, the final judgment will be made by the third researcher.

#### 3.7.2 Data extraction and management

The following information will be extracted from each study: the reference ID, the first author, year of publication, age and gender of patients, the number of included cases, randomization, allocation concealment method, blinding method, interventions and control measures, intervention group's sample size, intervention time, measure of outcome, primary outcomes, period of observation, follow-up duration, routine examination of safety, fund source and type. The researchers will contact the author of study in the case of insufficient reported data. If negotiation cannot help to come to agree on the extraction of data, the final judgment will come to a third researcher.

#### 3.7.3 Risk of bias assessment in included studies

Two researchers will independently adopt the Cochrane collaboration risk-of-bias assessment for assessing the quality of literature included in the review, together with completing the STRICTA checklist.^[29]^ Assessments include selective reporting, random sequence generation, incomplete outcome data, allocation concealment, blinding as well as other possible biases. Related standards proposed in the Cochrane Intervention System Assessment Manual will be considered to classify risk of bias into three levels: high risk, low risk, and unclear risk. The discrepancies will be resolved by discussion and the third author will make the final judgment if the two researchers cannot reach an agreement.

#### 3.7.4 Treatment effect measures

Odds ratio (OR) and risk ratio (RR) with 95% confidence interval (CI) will be used in measuring the treatment effects specific to dichotomous outcomes. For continuous outcomes, mean difference (MD) or standardized mean difference (SMD) with 95% CI will be calculated to measure the treatment effects.

#### 3.7.5 Unit of analysis issues

The study will use the data of patients in RCTs. Where more than one BJYST group is arranged in an RCT, multiple meta-analyses will be separately performed for each treatment group. Data of crossover trials will be obtained from the first sequence. The intervention and control groups will be analyzed by summarizing all controls’ results if there are many control measures.

#### 3.7.6 Missing data management

The reason for the loss of data missed in the period of data screening and extraction will be identified here. Corresponding author will be contacted for obtaining missing data. With missing data unable to be obtained, available data will be analyzed only and the reason and effect of such exclusion will be explained.

#### 3.7.7 Heterogeneity assessment

The meta-analysis will be carried out with the help of a random- or fixed-effects model. *Cochrane Handbook for Systematic Reviews of Interventions* describes that the Higgins’*I*^2^ statistic, a heterogeneity *x*^*2*^ test and a visual check of the forest plot can all help to assess the heterogeneity.^[21]^ A fixed-effects model will be used to pool the data with p value over 0.10 and the *I*^*2*^ value less than 50%. A random-effects model will be adopted in other cases. When a set of studies exhibit an obvious heterogeneity, factors leading to the heterogeneity will be discussed, like the characteristics of patients and the variation degree in interventions. The heterogeneity will be evaluated via the subgroup analysis or the sensitivity group if applicable.

#### 3.7.8 Reporting bias assessment

The biases of reporting will be assessed via virtue of a funnel plot if the meta-analysis includes over 10 trials. The asymmetry exhibited by the funnel plot will be evaluated via the Egger and Begger tests, and *P* value less than .05 will be considered that the publication bias is significant.

### 3.8 Data analysis

The RevMan 5 software (V. 5.3; Copenhagen: The Nordic Cochrane Centre, The Cochrane Collaboration, 2014) will be used for data analysis. The heterogeneity degree will help to confirm whether a random-effects model or a fixed-effects model will be used. The categorical variables will adopt the index of RR or OR and 95% CI. Continuous variables will adopt the index of MD or SMD and 95% CI. Chi-square distribution test and I2 statistic will be used to analysis the heterogeneity of included studies. If quantitative synthesis is not appropriate due to substantial heterogeneity, only qualitative analysis will be performed. The subgroup analysis shall carefully consider each subgroup in certain case. Meta-analysis will not be conducted if no assessment, like subgroup analysis, is able to explain existing meaningful heterogeneity.

### 3.9 Subgroup analysis

If necessary subgroup analysis and sensitivity analysis will be performed to explore possible causes of heterogeneity. Subgroup analyses will consider the heterogeneity exhibited by the following aspects: diopter, progress classification, astigmatism, the dosage of BJYST, duration of treatment, frequency of treatment, and measures in control groups. If quantitative synthesis is not applicable, we will proceed to narrative synthesis.

### 3.10 Sensitivity analysis

For testing, if review conclusions are robust, primary outcomes will receive a sensitivity analysis based on criteria involving the size of sample, the quality of heterogeneity and the statistic model (whether it is a random-effects model or a fixed-effects model).

### 3.11 Grading the evidence quality

The evidence quality for obtained results will be assessed via the GRADE method.^[30]^ The assessment includes risk of bias exhibited by studies, the heterogeneity, evidence directness, estimate precision of effect and publication risk of bias. Evidence will be divided into four categories considering the level: high risk, moderate risk, low risk, and very low risk.

### 3.12 Ethics and dissemination

Meta-analysis is a secondary study of the published data, thus there is no need for obtaining the ethical approval or patients’ informed consent. The results will be published in a peer-reviewed journal.

## 4 Discussion

Myopia is a relatively prevalent and increasing public health concern, particularly in East Asia, where it has already reached a pandemic level.^[31]^ Apart from the substantial socioeconomic cost, severe sight-threatening complications associated with high myopia substantially compromise quality of life.^[32]^ There are significant odds ratios (ORs) for myopic maculopathy, retinal detachment, cataracts, and glaucoma, even for low and moderate levels of myopia, and these ORs increase further with higher levels of myopia.^[33]^ Researchers and clinicians have proposed approaches to treat myopia for many years. However, to date, no ideal approach has been identified as efficacious for the prevention and treatment of myopia with sufficient safety and clinical acceptability.^[34]^

Chinese herbal medicines may be a reasonable and safe alternative to treat high myopia. Among the abundant Chinese herbal medicines, BJYST has been proven to be effective in the treatment of high myopia. However, its real efficacy and safety are not well understood. Therefore, this study will evaluate the efficacy and safety of BJYST in treating high myopia by searching and analyzing relevant studies comprehensively. In this study, we would draw a scientific conclusion and provide evidence of evidence-based medicine for BJYST in the clinical treatment of high myopia.

## Author contributions

**Conceptualization:** Jie Ma, Ya Mo.

**Data curation:** LinZhi Li, Jie Ma.

**Formal analysis:** Jie Ma.

**Methodology:** LinZhi Li, Jie Ma.

**Resources:** LinZhi Li, Jie Ma.

**Software:** LinZhi Li.

**Supervision:** Ya Mo.

**Writing – original draft:** LinZhi Li, Jie Ma.

**Writing – review & editing:** Ya Mo.
